# Social familiarity shapes collective decision-making in response to looming stimuli in Medaka fish

**DOI:** 10.1038/s41598-025-30656-4

**Published:** 2025-12-23

**Authors:** Ryohei Nakahata, Hideaki Takeuchi

**Affiliations:** 1 Molecular Ethology Laboratory, Graduate School of Life Science, Tohoku University, Sendai, 980-8577 Japan; 2 Department of Cardiac Regeneration Biology, National Cerebral and Cardiovascular Centre, Osaka, 564-8565 Japan

**Keywords:** Ecology, Ecology, Neuroscience, Zoology

## Abstract

Social familiarity within groups promotes behavioural synchrony and facilitates information transfer. Whether it shapes collective decision-making under predator threat is unknown. Here groups of six medaka (*Oryzias latipes*) familiarised for one month were used to test whether familiarisation promotes instantaneous collective decision-making in response to a looming stimulus (LS) mimicking a predator attack. First, we analysed behavioural transitions, defined as changes among three behavioural states: high-speed, normal and freezing-like before, during, and after LS in groups of six individuals. Individuals showing high-speed state in response to LS typically tended to shift to freezing-like state afterwards, whereas non-responders were more likely to maintain normal state. Group-level analysis revealed a bimodal distribution in the number of individuals exhibiting freezing-like state, with peaks at zero and six individuals, corresponding to ‘all non-freezing’ and ‘all-freezing’. Clustering analysis further identified three consistent group profiles: ‘freezing-dominant’, ‘non-freezing-dominant’, and ‘mixed-type’ based on behavioural tendencies across 10 trials. In contrast, in unfamiliar groups assembled immediately before testing, the ‘freezing-dominant’ profile was absent, and the distribution in the number of individuals exhibiting freezing-like state shifted to unimodal. In these groups, even at the individual level, responses more often showed a transition from high-speed state to normal state rather than freezing-like state. The results indicate that social familiarity promotes synchronous freezing-like state and consensus decisions under looming threat. Our study presents a behavioural assay for predator-evoked collective decision-making in a genetic model fish, providing a framework for future efforts to link behavioural ethology with neuroscience.

**Supplementary Information:**

The online version contains supplementary material available at 10.1038/s41598-025-30656-4.

## Introduction

Animals living in groups often exhibit synchronous behaviour in contexts such as migration, foraging, and predator avoidance. The emergence of coordinated behavioural patterns through interactions among individuals is termed collective behaviour, where group-level order and synchrony are thought to arise from local rules at the individual level, including attraction, alignment, and repulsion^1^. Collective behaviour includes collective decision-making, in which a group selects a single option from among multiple alternatives, and this phenomenon is observed in various contexts such as movement^2,3^, foraging^4^, and predator evasion^5–7^.

In the context of predator avoidance, consensus formation during collective decision-making has been studied across various species. For instance, in sticklebacks, once the number of individuals escaping in a particular direction exceeds a threshold, the remaining group members tend to follow^6^. Similarly, simulations involving humans have shown that when a critical number of escape responses are observed, the group tends to adopt avoidance behaviour^5^. In elephants, the oldest female has been reported to be particularly sensitive to predator vocalisations and to influence the group’s decision to flee^7^. However, few studies have quantitatively investigated collective decision-making as an instantaneous group response under emergency conditions. In particular, how dynamic group-level responses to a rapidly approaching predator emerge remains poorly understood. While mechanisms underlying rapid individual decision-making in response to visual looming stimuli have been demonstrated^8^, systematic analyses of dynamic group-level responses remain scarce.

Social familiarity has also been shown to affect individual recognition and interaction patterns, thereby influencing behaviour and information transmission^9^. At the dyadic level, social familiarisation enhances responsiveness to predators in predatory mites and reduces encounter frequency^10^. In sticklebacks, familiarisation reduced leadership tendencies in bold individuals, leading to more balanced coordination^11^. In cichlids, social familiarity promotes exploratory behaviour and reduces fear responses to novel stimuli^12^, while in zebrafish, the transmission of social fear is enhanced among familiar individuals^13^. At the group level, wild female guppies tend to associate with familiar individuals^14^, avoidance frequency in response to predator odour increases in fathead minnows^15^, and the latency to initiate avoidance of a predator model is reduced in brown trout^16^. In tropical damselfish, both responsiveness to fear stimuli and inter-individual information transmission are enhanced through familiarisation^17^. Overall, the literature indicates that social familiarity enhances alignment coordination and information transmission; however, how these factors influence collective decision-making remains unclear.

Furthermore, most empirical research in this field has relied on observations in natural environments or on wild individuals^6,15^, yet relatively few studies have been conducted in controlled experimental settings. Although collective decision-making regarding movement direction has been demonstrated in zebrafish^18^, reports of such decisions in response to predators are lacking. Moreover, integrative frameworks that link decision-making mechanisms at both the individual and group levels with molecular and neural analysis, particularly in genetically tractable model organisms, are still lacking.

To address these gaps, we focused on medaka *Oryzias latipes*, a well-established model organism in molecular genetics. Medaka are known to exhibit coordinated behaviour with conspecifics^19^ and to improve foraging efficiency through visual social learning^20^. Preliminary observations revealed that small groups of medaka responded synchronously to a human approach, either by showing ‘freezing after escape’ or by maintaining continuous movement without escape. Motivated by these findings, we aimed to develop a behavioural assay capable of quantitatively assessing instantaneous collective decision-making using a looming stimulus (LS) mimicking the sudden approach of a predator. LS has been used to elicit individual avoidance responses in mice^21^, zebrafish^22^, and fruit flies^23^. However, most previous studies have focused on individual-level decision-making^8^.Even in group contexts, the focus has remained on how individual responses are influenced by conspecifics^23,24^, with little attention paid to whether the group as a whole converges on a single collective choice.

In this study, we optimised LS parameters and established a quantitative behavioural system capable of replicating the distinct response patterns during preliminary observations. We then tested whether medaka groups exhibit instantaneous collective decision-making in response to LS and examined the effects of social familiarity on the decision-making patterns. Our findings establish an experimental model for examining collective decisions under acute threat. They also lay the foundation for elucidating how familiarisation modulates synchrony and group-level decision-making processes in a genetically accessible model organism.

## Methods

### Ethics statement

All the methods in this study were carried out in accordance with relevant guidelines and regulations. The work in this paper was conducted using protocols specifically approved by the Animal Care and Use Committee of Tohoku University (permit number: 2022LsA-003). All efforts were made to minimise suffering following the NIH Guide for the Care and Use of Laboratory Animals. Fish and breeding conditions are described above. The study was carried out in compliance with the ARRIVE guidelines (https://arriveguidelines.org/arrive-guidelines).

### Animals

**Fig. 1:**
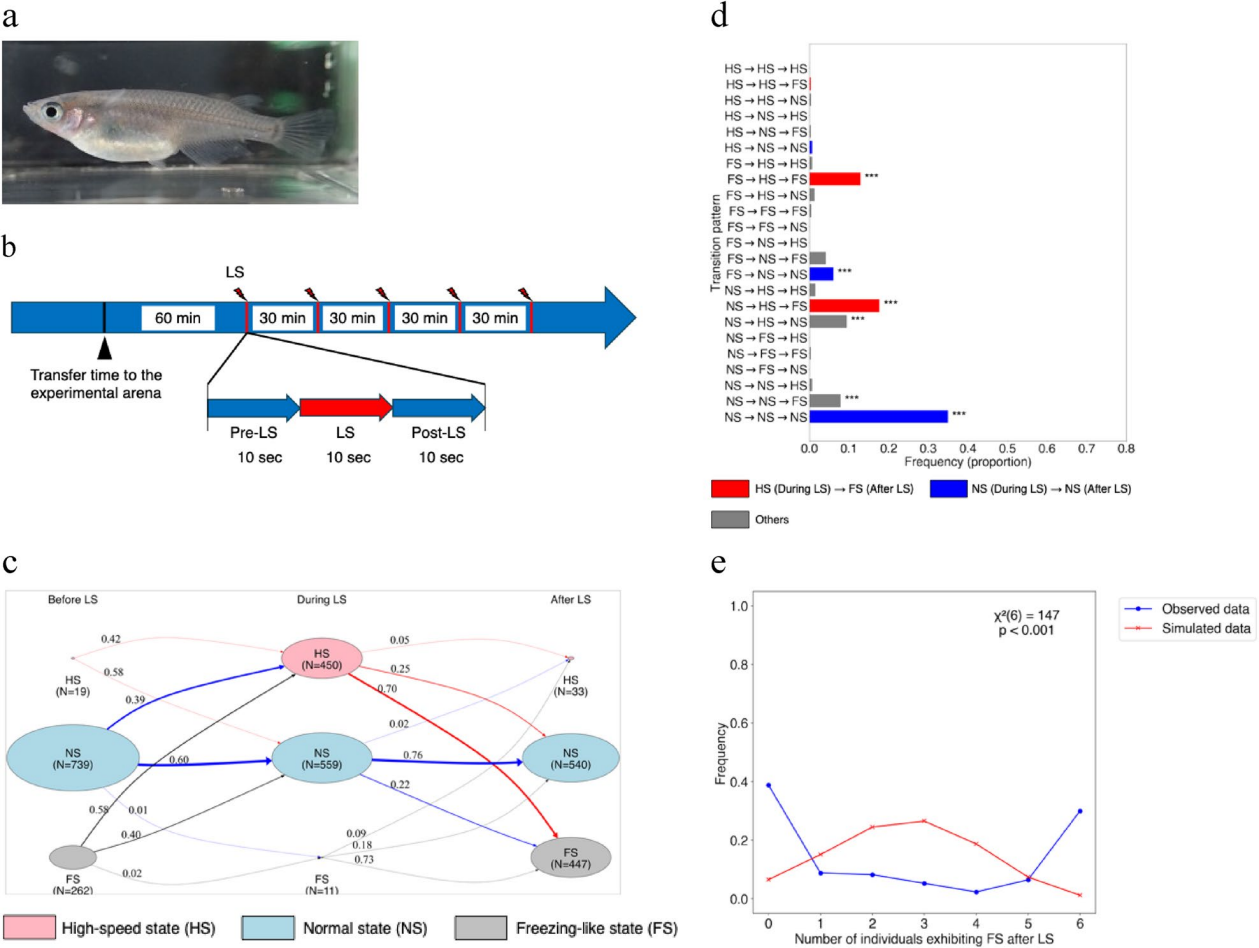
Experimental design, state transition analysis, and statistical evaluation of freezing-like behaviour in medaka after looming stimulus. (**a**) Medaka fish (fading strain) (**b**) Groups of medaka were transferred, with their tanks, from the circulating water system to the apparatus after feeding. One hour later, the looming stimulus (LS) was presented five times at 30-minute intervals. The experiment was conducted over two consecutive days, giving ten LS. For analysis, three 10-second periods (before, during, and after each LS) were used, totalling 30 s per trial. (**c**) A state transition diagram visualises individual-level states (FS: freezing-like state, NS: normal state, and HS: high-speed state) across three intervals: before, during, and after LS. Node size represents the proportion of individuals, and numbers within nodes indicate counts. Node colours are red for HS, light blue for NS, and grey for FS. Numbers on edges indicate the transition probabilities, and edge thickness corresponds to the number of individuals. Edge colours indicate the originating state: red for transitions from HS, blue from NS, and grey from FS. (**d**) A bar plot showing the frequencies of transition patterns across the three intervals. Each pattern is categorised according to the sequence of states. Red bars indicate transitions to HS during LS followed by FS after LS. Blue bars indicate individuals that remained NS during and after LS. Grey bars represent all other patterns. To assess statistical significance, a binomial test with false discovery rate (FDR) correction was applied. Patterns with *q* < 0.001 are marked with ***. (**e**) The X-axis shows the number of individuals in FS after LS, and the Y-axis shows its frequency. The blue and red lines represent the observed data (17 groups) and the simulated data (17 groups × 1000 trials, seed = 1, …, 1000), respectively. A chi-square test result is shown in the graph (χ² (6) = 147, *p* < 0.001).

Medaka (*Oryzias latipes*, fading strain) were obtained from Dr Tetsuro Takeuchi (Fig. 1a)^25^. This strain gradually loses body pigmentation in different body parts and at different timing among individuals, which initially appeared suitable for individual identification based on body colour. However, we later found that distinguishing individuals within a group of six remained difficult. During group observations, we consistently noted characteristic and reproducible collective behavioural patterns. As this strain has been maintained as a closed colony with limited genetic variation, we considered it an appropriate model for investigating the mechanisms underlying such stable group-level behavioural patterns. All individuals were hatched and bred in our laboratory. Medaka fish were maintained in groups of six individuals in plastic aquariums (22.6 cm× 14.6 cm× 14.5 cm, Sanko) or custom-made acrylic aquariums(22 cm × 14.5 cm × 14.5 cm) under controlled temperature (26 ± 1 °C) and light (14 h: 10 h light: dark) conditions. Every day, fish were fed brine shrimp between 12:00 and 13:00 and solid bait Otohime β−2 (Marubeni Nissin Feed, Tokyo, Japan) at least twice around 10:00 and 17:00 on weekdays. This study used individuals aged 2–9 months post hatch.

### Behavioural experiments under familiar conditions

Medaka (*Oryzias latipes*) aged either one month or nine months were randomly selected to form groups. Ten groups of six individuals each were formed from one-month-old fish (*N* = 60), and seven groups were formed from nine-month-old fish (*N* = 42). These groups were formed without regard to sex, because the sex ratio of one-month-old fish could not be reliably determined. In contrast, the nine-month-old groups were adjusted to achieve a 1:1 sex ratio. Each group of six individuals was maintained separately for a month. In total, 17 groups of sexually mature individuals aged either two months or ten months that had undergone familiarisation, were used for experiments.

### Behavioural experiments under unfamiliar conditions

Medaka aged 4 or 9 months, reared in a recirculating aquaculture system, were randomly selected. From the 4-month-old fish (*N* = 36), six groups were formed, and from the 9-month-old fish (*N* = 36), another six groups, each consisting of six individuals, yielded a total of 12 groups. The sex ratio in each group was adjusted to 1:1. Group formation took place in the morning, and experiments were conducted 1 to 2 h after the afternoon feeding. Subsequent procedures were conducted in accordance with those described in the familiarisation experiments.

### Looming stimulus (LS)

The looming stimulus (LS) was a visual stimulus mimicking an approaching predator, created using the “Zoom” animation function in Microsoft PowerPoint (Figure S1). The stimulus expanded to a width of 14.5 cm over 5.5 s, gradually darkened over 10 s, and remained black for 3 min (Figure S1). It was presented on an LCD monitor (EXLDH271DB, I-ODATA) mounted on the side of the aquarium (Figure S2). Both plastic and acrylic aquaria, also used as breeding tanks, were employed. Approximately 1–2 h after daytime feeding (12:00–13:00), each group was transferred in its breeding tank directly to the behavioural testing apparatus and the water level was adjusted to 6 cm. The LS was presented five times at 30-minute intervals for two consecutive days, beginning 1 h after transferring the aquarium from the rearing system to the testing apparatus (Fig. 1b).

### Behavioural recording and tracking

Behaviour was recorded from above using an action camera (M80 Air, Apexcam, or HERO8, GoPro) at a resolution and frame rate of 4 K (30 fps) or 2.7 K (50 fps). Recordings lasted 5 min, beginning 2 min before the LS and ending 3 min after. For analysis, a 30-s segment was extracted for each trial: 10 s before, during, and after LS (Fig. 1b). Video files were extracted using QuickTime Player, converted to JPEG format using FFMPEG (v4.4.1), and subsequently converted to MP4 format at 5 fps. Tracking was performed using UMATracker^26^, and coordinate data were obtained with the UMATracker-Tracking tool, applying either the Pochi-Pochi (manual positioning) or Group Tracker GMM algorithm. Tracking errors, such as identity swaps, were manually corrected using UMATracker-TrackingCorrector.

To convert pixel values to centimetres, the number of pixels along the centre of the long side of the aquarium was measured in ImageJ, which was based on the actual inner length (20.0–20.5 cm). Velocity (cm s⁻¹) was calculated from coordinate data (5 fps). A velocity matrix (6 individuals × 10 trials × 17 groups; 1020 × 150 frames) was compiled, and a moving average was applied (window size = 5) using pandas v1.4.0 to smooth short-term fluctuations.

### Definition of behavioural types and states

To capture how individual fish responded to LS (looming stimulus) in terms of state transitions, we expressed behavioural responses as transition patterns across three intervals (before, during, and after LS). In total, 17 groups of six individuals each (*N* = 102) were tested. For each group, fish were transferred to the experimental arena and habituated for one hour, after which the looming stimulus was presented five times at 30-minute intervals over two consecutive days (Fig. 1b). This protocol yielded a total of 6 individuals × 10 trials × 17 groups of individual-level datasets for subsequent analyses. For this purpose, we first defined behavioural types for each interval based on velocity data. Using histograms and kernel density estimation (KDE) curves of velocity, we categorised behaviour into three types: ‘freezing-like behaviour’, ‘normal swimming’, and ‘high-speed swimming’. The rationale for these definitions is as follows. Some individuals exhibited freezing-like behaviour after LS. The velocity histogram for the post-LS interval showed a bimodal distribution with a trough at approximately 0.2 cm/s (Figure S3a). Therefore, frames with speeds below 0.2 cm/s were defined as ‘freezing-like behaviour’. During LS, escape-like responses characterised by high-speed swimming were observed. Such behaviours were rarely seen before the LS onset. Comparison of KDE curves for the pre-LS and LS intervals revealed minimal overlap above 6 cm/s (Figure S3b). Thus, frames with speeds of 6 cm/s or higher were defined as ‘high-speed swimming’. Frames with velocities between 0.2 cm/s and 6 cm/s were categorised as ‘normal swimming’. All histograms, KDE curves, and heat maps were generated using Python v3.8 and matplotlib v3.7.5.

Based on speed-based behavioural types, we then defined the behavioural state for each 10-second interval (before LS, during LS, and after LS). An interval was categorised as a ‘freezing-like state (FS)’ if freezing-like behaviour persisted for ≥ 8 s, and as a ‘normal state (NS)’ if freezing-like behaviour lasted for < 2 s. During LS, escape behaviour occurred rapidly. Therefore, if high-speed swimming (≥ 6 cm/s) was sustained for 0.2 s (equivalent to one frame at 5 fps), we defined this as a ‘high-speed state (HS)’. This threshold reflects the minimum temporal resolution required to identify continuous motion.

### Statistical analysis

#### Characterisation of state transition patterns at the individual level

To calculate the state transition probabilities for behavioural transitions before, during, and after LS, we constructed state transition matrices by counting the number of transitions between states and normalising each row, following established methods using Markov chain analysis^27,28^. To visualise the transition dynamics, we created state transition diagrams using python-graphviz v0.20.3, where each state (NS, FS, and HS) was represented by a node, with edges indicating transition probabilities.

Furthermore, we used a binomial test to compare whether there were significantly more specific state transition patterns in the series of flows from before LS to after LS. In the binomial test, we set the null hypothesis that ‘the 27 behavioural patterns occur with equal probability (1/27)’ and performed a one-sided test. To control for type I errors due to multiple comparisons, we applied FDR correction to the binomial test results.

#### Analysis of group-level freezing-like states after LS

Based on the analysis of individual-level behavioural patterns, we next examined group-level freezing-like states (FS) after LS. For each trial, the number of individuals in the FS after LS was counted. To test whether synchronous FS occurred, virtual datasets were generated by randomly shuffling the states of each trial among groups (17 groups × 1,000 trials; seeds = 1, 2, …, 1,000). The proportions of individuals in each state across all trials were compared between the virtual datasets and the observational data using a chi-square test (scipy v1.10.1). The null hypothesis was defined as: “The presence or absence of the FS for each individual is independent, and synchronous FS for the entire group occur at random.”

#### Classification of group response profiles

To classify these characteristics, we performed a principal component analysis (PCA) on 27 individual-level behavioural patterns from before to after the LS intervention. We then calculated the cumulative contribution rate and reduced the number of dimensions to the minimum required to explain > 95% of the variance. To visualise the cluster structure, we further projected the PCA-reduced data using UMAP^29^ (umap-learn v0.5.7). Classification was performed using spectral clustering (scikit-learn v1.2.2), and the optimal number of clusters was determined based on the silhouette coefficient. This coefficient approaches 1 when intra-cluster cohesion and inter-cluster separation are high; therefore, the number of clusters yielding the highest silhouette coefficient was selected.

#### Comparison of state transition patterns at the individual level between clusters

Differences in the frequency of state transition patterns between clusters were evaluated using binomial tests with FDR correction, as described above.

#### Analysis of group-level freezing-like states

To examine differences in group-level freezing-like states (FS) across clusters and between familiar and unfamiliar groups after LS, we applied a generalized linear mixed model (GLMM)^30^. The dependent variable was the number of individuals exhibiting the FS (0–6) within each group. Cluster identity and the presence or absence of familiarisation were included as fixed effects. Experimental group identity, trial number, and group identity were incorporated as random effects to account for repeated measurements and inter-group variability. The model assumed a binomial distribution with a logit link function, which is appropriate for categorical or count data with hierarchical structure. Analyses were performed in Python v3.8.12. We used pyper v1.1.2 to call R v4.1.2, and the lme4 and multcomp packages for model fitting and post hoc tests. Tukey’s method was applied for multiple comparison correction.

## Results

### Detection of individual-level state transition characteristics

To examine how individuals responded to the looming stimulus (LS), we analysed behavioural transitions across three intervals: before, during, and after LS. Fish behaviour was first classified into three types based on swimming velocity: freezing-like (< 0.2 cm/s), normal (0.2–6 cm/s), and high-speed (≥ 6 cm/s). Each 10-second interval was then categorized into one of three behavioural states—freezing-like, normal, or high-speed—according to duration thresholds (≥ 8 s freezing, < 2 s freezing, and ≥ 0.2 s high-speed). These definitions enabled consistent identification of state transitions across trials. Using these thresholds, each of the three temporal intervals was classified into one of the three behavioural states: ‘freezing-like state (FS)’, ‘normal state (NS)’, or ‘high-speed state (HS)’ (Figure S4).

To examine how the behavioural states of individuals transitioned from before LS to during LS, and from during LS to after LS, we calculated state transition probabilities using a Markov chain and visualised them as a state transition diagram (Fig. 1c). Between the pre-LS and LS intervals, 39% of individuals transitioned from NS to HS, whereas 60% remained in NS. Among individuals that entered HS during LS, 70% transitioned to FS after LS. In contrast, individuals that remained in the NS during LS had a 76% probability of continuing in that state after LS. These findings suggest that individuals showing escape-like behaviour during LS tended to transition into FS after LS, whereas those unresponsive to LS generally maintained NS.

To statistically evaluate trends in state transition patterns, we extracted behavioural state sequences across the three intervals (before, during, and after LS). Each sequence was expressed as a combination of the three defined behavioural states: HS, NS, and FS, resulting in 27 possible transition patterns. We quantified the frequency of each pattern across trials (Fig. 1d).

The most frequent transition pattern was the maintenance of NS throughout the three intervals (NS→NS→NS; blue; *q* < 0.001). The second most frequent pattern was NS→HS→FS (red; *q* < 0.001), and the third was FS→HS→FS (red; *q* < 0.001), both involving a transition to HS during LS followed by FS: NS→HS→FS (red; *q* < 0.001) and FS→HS→FS (red; *q* < 0.001). Additional significantly overrepresented patterns were NS→HS→NS (grey; *q* < 0.001), NS→HS→FS (grey; *q* < 0.001), and FS→NS→NS (blue; *q* < 0.001), all of which exceeded the expected frequency under a uniform distribution (1/27).

Among these six prominent transition patterns (Fig. 1d), NS→NS→NS and FS→NS→NS represent non-reactive behaviours where individuals maintained or returned to the NS during and after LS (blue). In contrast, NS→HS→FS and FS→HS→FS represent reactive responses, characterised by HS during LS followed by FS (red). These two reactive patterns accounted for 41% and 30% of all observations, respectively, and thus constitute the typical individual-level responses to LS stimulation. Notably, escape without subsequent FS (NS→HS→NS; approx. 9%) and no initial response followed by FS after LS (NS→NS→FS; approx. 8%) were also observed at appreciable frequencies.

### Population polarisation into synchronous freezing-like and non-freezing-like states after LS

To investigate whether medaka groups exhibited synchronous responses (either FS or non-FS) after LS, we counted the number of individuals exhibiting FS in each trial. The distribution was bimodal, with peaks at 0 and 6 individuals (Fig. 1e, blue line), suggesting that entire groups tended to respond uniformly.

To determine whether this distribution could be explained by chance, we generated a virtual dataset by randomly shuffling the individual-level FS and non-FS classifications within groups of the same size (Fig. 1e, red line). This reconstructed the expected distribution under the assumption that individuals responded independently of one another. A chi-square test comparing the observed and expected distributions revealed a significant difference (χ² (6) = 147, *p* < 0.001). This result suggests that the strong bias towards either ‘all-freezing’ or ‘all non-freezing’ within groups after LS is unlikely to have occurred by chance alone. Instead, it indicates that individuals within a group reacted in a synchronous manner through social interaction.

### Group response profiles to LS classified into three types

During the behavioural experiments, we observed groups in which all individuals synchronously exhibited FS, as well as groups in which all individuals remained unresponsive and continued swimming. Moreover, the same groups tended to display similar response tendencies across repeated trials. Based on these preliminary observations, we hypothesised that groups exhibit consistent and characteristic behavioural tendencies, which we define as group response profiles. To evaluate this hypothesis, we classified groups according to their individual-level behavioural patterns. Specifically, behavioural data from 10 trials per group were aggregated, dimensionality reduction was performed using principal component analysis (PCA) followed by UMAP, and spectral clustering was applied to classify the groups.

**Fig. 2:**
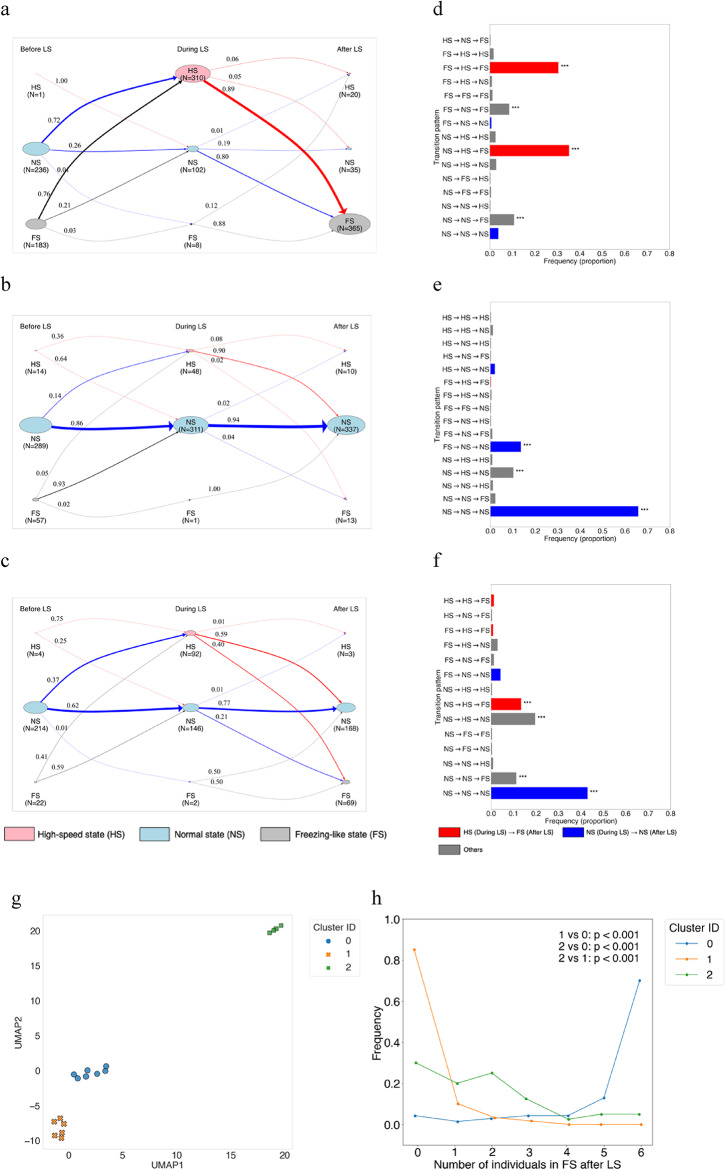
State transition diagrams and frequencies of behavioural state transition patterns for each cluster. (**a–c**) State transition diagrams for each cluster were generated to represent individual-level behavioural states (FS: freezing-like state, NS: normal state, and HS: high-speed state) before, during, and after LS. All the visual elements are consistent with those in Fig. 1c. (**a**) State transition diagram for Cluster 0. b) State transition diagram for Cluster (1) (**c**) State transition diagram for Cluster (2) (**d–f**) Bar graphs showing the frequency of occurrence for each behavioural state transition pattern in each cluster. Details of colour coding and statistical tests are as described in Fig. 1d. (**d**) Distribution of transition pattern frequencies in Cluster 0. (**e**) Distribution of transition pattern frequencies in Cluster (1) (**f**) Distribution of transition pattern frequencies in Cluster (2) (**g**) The X-axis represents the first UMAP component and the Y-axis the second. Each point shows the group centroid, obtained by reducing the original 23 dimensions to 16 dimensions using PCA and further to two dimensions using UMAP. Colours indicate classification results based on spectral clustering. (**h**) The X-axis represents the number of individuals in the freezing-like state after LS, and the Y-axis represents the frequency of these counts across all trials. The lines correspond to the IDs of the three clusters. Tukey’s post hoc test based on a GLMM was performed, and the results of the cluster comparisons are shown within the graph.

PCA indicated that 16 dimensions were required to exceed a cumulative variance contribution of 95%, and this was adopted as the optimal dimensionality (Figure S5). The reduced data were then projected into two dimensions using UMAP, revealing a clear distinct group-level structures (Fig. 2g). Spectral clustering, guided by the silhouette coefficient identified three as the optimal number of clusters (Figure S6). Accordingly, groups were classified into three clusters (Figure S7).

To analyse state transitions of individuals across the pre-, during-, and post-LS intervals in each cluster, we calculated state transition probabilities using a Markov chain and visualised them as state transition diagrams (Fig. 2a-c).

In Cluster 0 (Fig. 2a), the probability of transitioning from NS to HS from before LS to during LS was 72.5%, while the transition probability from FS to HS was 76%. Individuals that exhibited HS during LS had an 89% probability of subsequently transitioning to the FS. In addition, even individuals that remained in NS during LS had an 80.4% probability of transitioning to FS afterwards. These results suggest that in Cluster 0, both responsive (HS) and unresponsive (NS) individuals tended to synchronise into a FS after LS. The most frequent pattern was NS→HS→FS (red; *q* < 0.001), and the second was FS→HS→FS (red; *q* < 0.001), both involving a transition to HS during LS followed by FS (Fig. 2d). These findings suggest that Cluster 0 corresponds to groups in which all individuals synchronise and exhibit FS after LS.

In Cluster 1 (Fig. 2b), the probability of maintaining the NS from before LS to during LS was 86.2%, and individuals that remained unresponsive during LS continued NS after LS with a probability of 94.2%. The dominant patterns were those where individuals maintained NS throughout (NS→NS→NS and FS→NS→NS, *q* < 0.001; Fig. 2e, blue). These results suggest that Cluster 1 corresponds to groups in which all individuals synchronise and maintain NS after LS.

In Cluster 2 (Fig. 2c, f), NS→NS→NS remained the most frequent pattern (*q* < 0.001). However, various other transitions were also observed, including NS→HS→NS (*q* < 0.001; grey), NS→HS→FS (*q* < 0.001; red), and NS→NS→FS (*q* < 0.001; grey). This diversity of transitions indicates that Cluster 2 represents a heterogeneous group response profile, reflecting a mixture of multiple individual-level response types rather than a single dominant pattern.

### Synchronisation of freezing-like and non-freezing-like states across the group profiles

Individual-level state transition analysis revealed that in Cluster 0, individuals frequently responded to the looming stimulus (LS) and then entered FS, whereas in Cluster 1, transitions in which individuals did not respond to LS and continued NS were predominant. We next examined whether all individuals in Cluster 0 synchronised to exhibit FS, and whether all individuals in Cluster 1 synchronised to continue NS. To this end, we counted the number of individuals in FS after LS for each group and compared these counts across clusters. A significant difference was observed in the number of individuals exhibiting FS (Fig. 2h, *p* < 0.001). In Cluster 0, the most frequent outcome was that all six individuals showed FS, whereas in Cluster 1, the most likely outcome was that no individual showed FS. In Cluster 2, the number of individuals exhibiting FS ranged mostly from zero to three, yielding a distribution distinct from both Clusters 0 and 1. These results indicate that in Cluster 0, individuals tended to synchronise to FS, whereas in Cluster 1 they synchronised to non-FS (continued NS). In contrast, Cluster 2 showed no clear synchronisation, with only a subset of individuals exhibiting FS after LS.

### Individual-level differences in behavioural transition patterns between familiar and unfamiliar groups

**Fig. 3:**
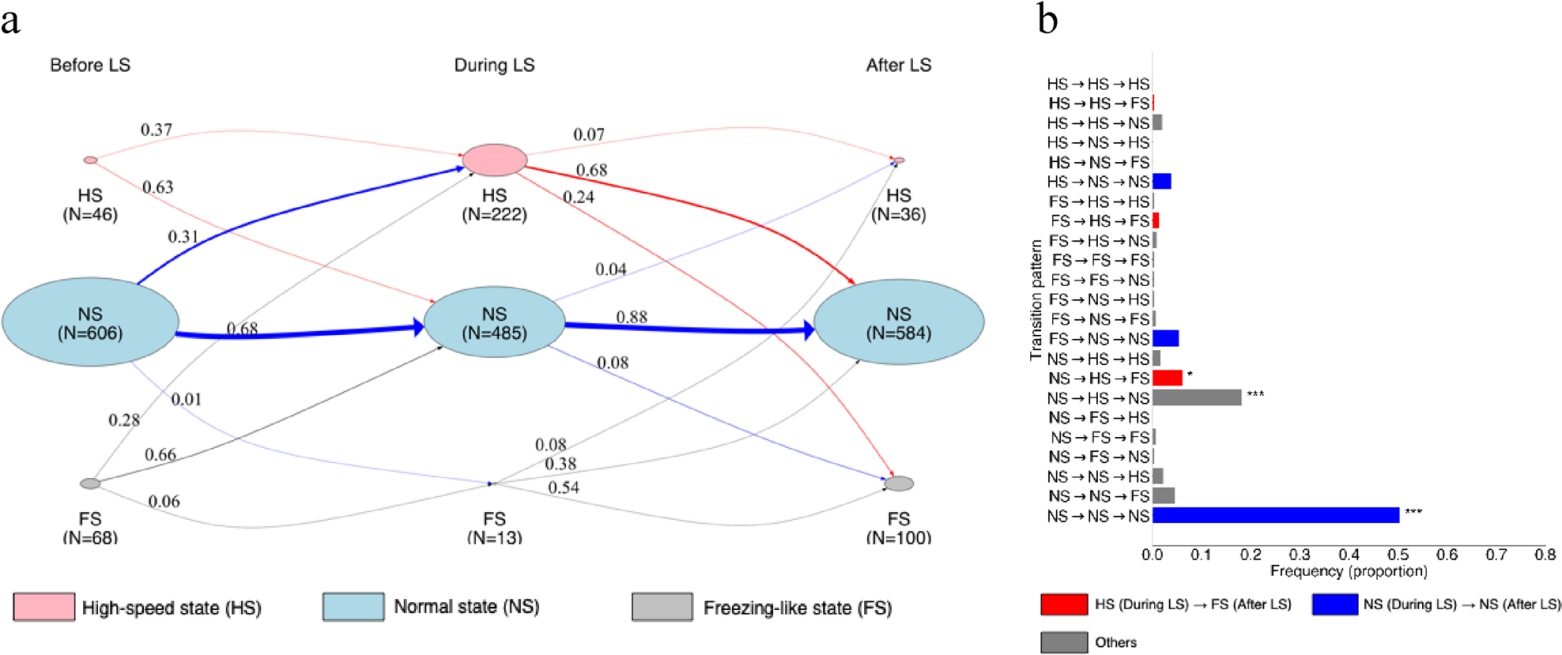
State transition diagram of individual-level behaviour (Unfamiliar group). (**a**) For individual-level behaviours classified as FS: freezing-like state, NS: normal state, or HS: high-speed state, the state transition probabilities were shown from before to during LS and from during to after LS using a Markov chain. Details are as described in Fig. 1c. (**b**) Bar graphs showing transition patterns and their frequencies from before LS to during and after LS for FS, NS, and HS. Statistical significance is denoted as follows: ***q < 0.001, **q < 0.01, *q < 0.05, and no notation for *q* > 0.05. Details are as described in Fig. 1d.

We determined the behavioural patterns of individuals in the unfamiliar group (Figure S7-8), constructed a state transition diagram (Fig. 3a), and classified individual-level transition patterns into 27 categories, comparing their frequencies of occurrence (Fig. 3b). As in the familiar groups, the most frequent pattern was the non-reactive type (NS→NS→NS, blue; *q* < 0.001). The pattern (NS→HS→NS, grey) in which fish transitioned to HS during LS and returned to NS afterwards also appeared at a significantly high frequency (*q* < 0.001). In addition, the pattern in which fish transitioned to HS during LS and then entered FS after LS (NS→HS→FS, red) occurred significantly more often (*q* < 0.05). However, the patterns in which individuals entered FS after LS (NS→HS→FS, red; NS→NS→FS, grey), which were significantly enriched in the familiar groups, did not reach significance in the unfamiliar group. These findings suggest that individual-level transition patterns differed between the two conditions.

### Absence of group-level synchronous freezing-like state in the unfamiliar group

To test whether individuals exhibited synchronous responses after LS, we counted the number of individuals in FS per trial for each group and compared the observed data with control data generated by virtual shuffling, as in the familiar groups (Figure S9). In the observed data, the number of freezing-like individuals peaked at zero, and there was a significant difference in both the number and frequency of freezing-like individuals between the observed and virtual data (χ² (6) = 22.1, *p* < 0.01) (Figure S9). These results indicate that the peak at zero was not coincidental, but rather that all individuals within the unfamiliar group tended to exhibit synchronous non-FS, continuing to NS after LS.

### Differences in synchronous freezing-like states between familiar and unfamiliar groups

To examine whether the occurrence of FS at the group level after LS differed depending on familiarisation, the number of individuals exhibiting FS per trial was counted for each group. The aggregated group-level data were then compared between the familiar and unfamiliar groups using GLMM (Figure S10). The results showed that, following LS, the familiar groups tended to have a higher number of individuals exhibiting FS (Figure S10, *β* = 2.02, *p* < 0.05) and displayed a bimodal distribution. In contrast, the unfamiliar group showed fewer freezing-like individuals and exhibited a unimodal distribution. This indicates that, unlike the familiar groups, the unfamiliar group lacked the peak where all six individuals exhibited FS after LS.

### Disappearance of the collective freezing in the unfamiliar group

**Fig. 4:**
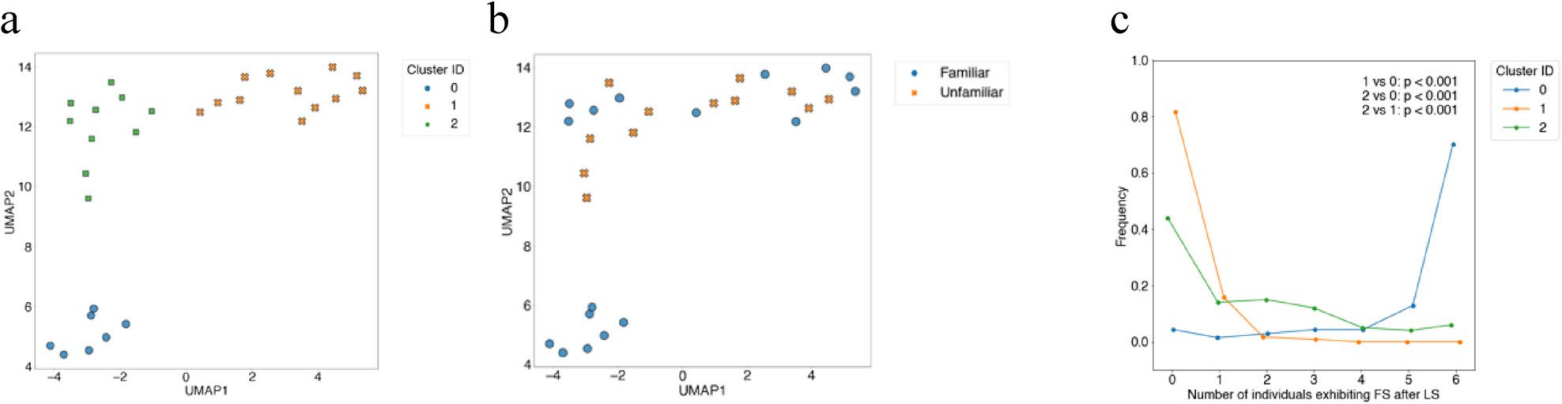
Visualisation of dimensionality reduction using PCA and UMAP, and comparison of freezing-like states across clusters. (**a–b**) The X-axis represents the first UMAP component and the Y-axis the second. Each point corresponds to the group centroid. (**a**) Colours indicate cluster IDs obtained by spectral clustering. (**b**) Colours indicate familiarisation status: familiar groups (blue) and unfamiliar groups (orange). (**c**) Distribution of freezing-like states (FS) after LS exposure in the integrated dataset combining familiar and unfamiliar groups. Details are as described in Fig. 2h.

To clarify similarities and differences in collective behavioural patterns between familiar and unfamiliar groups, we integrated and analysed data from both conditions. Specifically, we performed dimensionality reduction and clustering based on 27 individual-level behavioural transition patterns. Principal component analysis (PCA) revealed that 17 dimensions were required to explain 95% of the variance, which was therefore set as the optimal number (Figure S11). The silhouette coefficient indicated that the optimal number of clusters was three (Figure S12). The clustering results were visualised using a two-dimensional UMAP embedding derived from the 17 principal components and classified into three clusters by spectral clustering (Fig. 4a). Groups in the familiar condition were distributed across all three clusters, whereas the unfamiliar groups were absent from Cluster 0, indicating a clear bias (Fig. 4b). We next verified that in Cluster 0, all individuals tended to exhibit synchronous FS, while in Cluster 1 they tended to exhibit synchronous non-FS (continued NS). In Cluster 2, synchrony was absent, with only a subset of individuals showing FS after LS. Importantly, the classification showed that the unfamiliar groups were not represented in Cluster 0, showing that the ‘freezing-dominant’ cluster was absent from their collective behavioural profiles (Fig. 4c). To evaluate whether this disappearance of the freezing-dominant response could be attributed to the immediate formation of unfamiliar groups, we compared the distributions of the number of freezing-like individuals between groups tested on the first and the following day. Although a significant difference was detected (χ² (6) = 14.2, *p* = 0.028), this was mainly due to a slight increase in groups with two or four freezing-like individuals, whereas the frequency of ‘all-freezing’ remained almost unchanged (Figure S13). These results suggest that handling or grouping stress immediately after formation had little effect, and that the disappearance of freezing-dominant is a robust feature of unfamiliar groups.

## Discussion

In this study, we established a quantitative behavioural assay to analyse collective decision-making in medaka (O*ryzias latipes*) in response to a looming stimulus (LS). In this system, small groups of medaka were presented with an LS that mimicked an approaching predator, and their collective behavioural choices were examined. Two dichotomous collective response patterns consistently emerged at the group level: ‘all-freezing’ and ‘all non-freezing’. Furthermore, the distribution of the number of FS individuals per trial was bimodal, with clear peaks at either zero or six individuals. These results demonstrate the presence of a dichotomous collective behavioural choice in medaka and validate as a robust tool for investigating collective decision-making under controlled laboratory conditions. Moreover, this assay will provide a platform for elucidating the genetic and neural bases of collective decision-making in vertebrates.

Previous studies of collective decision-making under laboratory conditions have primarily used small fish species, such as sticklebacks and golden shiners^2,4,6^, often focusing on wild populations in ecological contexts. By contrast, our study employed medaka, a well-established genetic model organism, thereby enabling experimental systems in which genetic and environmental factors can be controlled. This approach enables the establishment of highly reproducible behavioural assays and allows for long-term monitoring of behavioural development, from the individual to the group level.

Most previous studies of collective decision-making have focused on gradual responses to predators^6,7^. In contrast, our findings revealed a novel phenomenon: rapid collective responses to sudden visual threats. In coral reef fishes, escape responses of individuals can be predicted from the expansion rate of looming stimuli or the behaviour of neighbours^24^. However, how these individual responses converge into synchronous group-level behaviour remains unclear. Our results demonstrate that under time-constrained predatory threat, rapid collective decision-making can emerge, thereby complementing existing models of gradual escape behaviour.

Our findings further suggest that a certain period of familiarisation is required for collective behavioural choices in response to the LS. In particular, in familiar groups, many individuals transitioned from HS during LS to FS afterwards, and entire groups tended to enter the FS. Such consistent behavioural synchrony was mainly observed in groups that had undergone sufficient familiarisation, suggesting that social familiarity may contribute to coordinated collective decisions. In our experiments, social familiarity increased the proportion of individuals that exhibited a FS after escape-like HS, indicating a change in behavioural regularity at the individual level. However, these individual-level changes alone cannot fully explain the emergence of dichotomous collective outcomes (all-freezing versus all non-freezing). It remains unclear whether familiarisation (1) enhanced each individual’s social sensitivity, making them more likely to be influenced by others, or (2) homogenised behavioural traits within groups. Distinguishing between these two possibilities was beyond the scope of this study. Future approaches incorporating longitudinal tracking of individually identified fish and quantitative measures of behavioural synchrony will be necessary to address this question.

If explanation (1) is correct, repeated interactions during familiarisation may allow individuals to recognise and predict the behaviour of conspecifics, thereby strengthening group-level properties such as polarisation and alignment. Previous studies have reported various effects of social familiarity in fish. For example, in female guppies, 12 days of familiarisation led to preferential associations with familiar conspecifics^31^. Social familiarity has also been shown to promote group cohesion and alignment in guppies^14^ and to enhance information transfer under social threat in damselfish^17^. In addition, familiarisation may also induce social fear contagion. In zebrafish, individuals are known to switch from high-speed swimming to freezing when exposed to alarm cues from conspecific skin extracts^13,32^, suggesting that this behavioural pattern may be widespread among fishes. Moreover, zebrafish exhibit similar freezing-like responses when observing familiar conspecifics or groups displaying fear responses^13,33^.

On the other hand, explanation (2), that familiarisation homogenises behavioural traits within groups, cannot be excluded. Previous studies have shown that bold individuals tend to maintain stable behavioural traits, whereas shy individuals are more plastic and influenced by social context. For instance, in guppies, bold individuals rely on their own information and explore independently, whereas shy individuals adjust their behaviour according to social information^34^. In sticklebacks, bold individuals also show stable exploratory behaviour, while shy individuals display behavioural plasticity and can change over time^35^. However, to our knowledge, no studies have directly demonstrated long-term homogenisation of behavioural traits caused by familiarisation in any animal species. Thus, we consider explanation (1) to be the more plausible mechanism underlying our findings.

Our study therefore extends previous fish familiarity research, which has reported average increases in shoal cohesion, by showing that long-term social familiarity also structures the variability of collective decisions. Familiar groups not only became cohesive; they also differentiated into groups that reached full consensus (freezing-dominant or non-freezing-dominant) and groups that failed to do so (mixed-type), revealing that familiarity regulates the probability—rather than the inevitability—of consensus formation under threat.

In summary, our results indicate that social familiarity promotes the dichotomisation of collective behavioural choices in medaka, and that factors such as social familiarity or changes in social sensitivity may contribute to this process. Although further investigation will be required to directly verify these mechanisms, our study provides a foundation for exploring how social experience shapes collective decision-making under time-constrained predatory threats in vertebrates.

## Supplementary Information

Below is the link to the electronic supplementary material.

Supplementary Material 1

## Data Availability

All data generated or analysed during this study are available from the corresponding author on reasonable request.

## References

[1] Krause, J. & Ruxton, G. D. in *Living in Groups*. (eds Krause, J. & Ruxton, G. D.) (Oxford University Press, 2002). 10.1093/oso/9780198508175.002.0001

[2] Couzin, I. et al. Uninformed individuals promote democratic consensus in animal groups. *Science* **334**, 1578–1580 (2011). 22174256 10.1126/science.1210280

[3] Strandburg-Peshkin, A., Farine, D. R., Couzin, I. D. & Crofoot, M. C. Shared decision-making drives collective movement in wild baboons. *Science* **348**, 1358–1361 (2015). 26089514 10.1126/science.aaa5099 PMC4801504

[4] Ward, A. J., Krause, J. & Sumpter, D. J. Quorum decision-making in foraging fish shoals. *PloS One*. **7**, e32411 (2012). 22412869 10.1371/journal.pone.0032411 PMC3296701

[5] Clément, R. J. G., Wolf, M., Snijders, L., Krause, J. & Kurvers, R. H. J. M. Information transmission via movement behaviour improves decision accuracy in human groups. *Anim. Behav.* **105**, 85–93 (2015).

[6] Ward, A. J. W., Sumpter, D. J. T., Couzin, I. D., Hart, P. J. B. & Krause, J. Quorum decision-making facilitates information transfer in fish shoals. *Proc. Natl. Acad. Sci.* **105**, 6948–6953 (2008). 18474860 10.1073/pnas.0710344105 PMC2383955

[7] McComb, K. et al. Leadership in elephants: the adaptive value of age. *Proc. R Soc. B Biol. Sci.* **278**, 3270–3276 (2011). 10.1098/rspb.2011.0168 PMC3169024 21411454

[8] Shang, C. et al. Divergent midbrain circuits orchestrate escape and freezing responses to looming stimuli in mice. *Nat. Commun.* **9**, 1232 (2018). 29581428 10.1038/s41467-018-03580-7 PMC5964329

[9] Ward, A. J. W. & Hart, P. J. B. The effects of kin and familiarity on interactions between fish. *Fish. Fish.* **4**, 348–358 (2003).

[10] Strodl, M. A. & Schausberger, P. Social familiarity reduces reaction times and enhances survival of group-living predatory mites under the risk of predation. *PLOS ONE*. **7**, e43590 (2012). 22927997 10.1371/journal.pone.0043590 PMC3425479

[11] Riley, R. J., Kwon, Y. M., Manica, A. & Savage, J. L. Familiarity dampens the effect of boldness on coordination in three-spined sticklebacks. *Behaviour* **162**, 191–206 (2025).

[12] Galhardo, L., Vitorino, A. & Oliveira, R. F. Social familiarity modulates personality trait in a cichlid fish. *Biol. Lett.* **8**, 936–938 (2012). 22859562 10.1098/rsbl.2012.0500 PMC3497109

[13] Fernandes Silva, P., Garcia de Leaniz, C. & Luchiari, A. C. Fear contagion in zebrafish: a behaviour affected by familiarity. *Anim. Behav.* **153**, 95–103 (2019).

[14] Davis, S., Lukeman, R., Schaerf, T. M. & Ward, A. J. W. Familiarity affects collective motion in shoals of guppies (*Poecilia reticulata*). *R Soc. Open. Sci.* **4**, 170312 (2017). 28989737 10.1098/rsos.170312 PMC5627077

[15] Chivers, D., Brown, G. & Smith, J. Familiarity and shoal cohesion in Fathead minnows (*Pimephales promelas*): implications for antipredator behaviour. *Can. J. Zool.* **73**, 955–960 (1995).

[16] Griffiths, S., Brockmark, S., Höjesjö, J. & Johnsson, J. Coping with divided attention: the advantage of familiarity. *Proc. Biol. Sci.* **271**, 695–699 (2004). 15209102 10.1098/rspb.2003.2648 PMC1691656

[17] Nadler, L. E., McCormick, M. I., Johansen, J. L. & Domenici, P. Social familiarity improves fast-start escape performance in schooling fish. *Commun. Biol.* **4**, 897 (2021). 34285330 10.1038/s42003-021-02407-4 PMC8292327

[18] Kadak, K. & Miller, N. Follow the straggler: zebrafish use a simple heuristic for collective decision-making. *Proc. R. Soc. B Biol. Sci.* 287:20202690 (2020). 10.1098/rspb.2020.2690 PMC7739921 33259757

[19] Imada, H. et al. Coordinated and cohesive movement of two small conspecific fish induced by eliciting a simultaneous optomotor response. *PLoS One*. **5**, e11248 (2010). 20582314 10.1371/journal.pone.0011248 PMC2889830

[20] Ochiai, T., Suehiro, Y., Nishinari, K., Kubo, T. & Takeuchi, H. A new data-mining method to search for behavioral properties that induce alignment and their involvement in social learning in Medaka fish (*Oryzias latipes*). *PLoS One*. **8**, e71685 (2013). 24039720 10.1371/journal.pone.0071685 PMC3765494

[21] Yilmaz, M. & Meister, M. Rapid innate defensive responses of mice to looming visual stimuli. *Curr. Biol.* **23**, 2011–2015 (2013). 24120636 10.1016/j.cub.2013.08.015 PMC3809337

[22] Temizer, I., Donovan, J. C., Baier, H. & Semmelhack, J. L. A visual pathway for looming-evoked escape in larval zebrafish. *Curr. Biol.* **25**, 1823–1834 (2015). 26119746 10.1016/j.cub.2015.06.002

[23] Ferreira, C. H. & Moita, M. A. Behavioral and neuronal underpinnings of safety in numbers in fruit flies. *Nat. Commun.* **11**, 4182 (2020). 32826882 10.1038/s41467-020-17856-4 PMC7442810

[24] Hein, A. M., Gil, M. A., Twomey, C. R., Couzin, I. D. & Levin, S. A. Conserved behavioral circuits govern high-speed decision-making in wild fish shoals. *Proc. Natl. Acad. Sci.* **115**, 12224–12228 (2018). 30420510 10.1073/pnas.1809140115 PMC6275531

[25] Takeuchi, T. & Manabe, E. Genetical study on the new mutant of the fading medaka, *Oryzias latipes*. *Res. Bull. Shujitsu Women’s Coll. Shujitsu Jr Coll.* **14**, 1–18 (1984).

[26] Yamanaka, O. & Takeuchi, R. UMATracker: an intuitive image-based tracking platform. *J. Exp. Biol.* **221**, jeb182469 (2018). 29954834 10.1242/jeb.182469

[27] Tuqan, M. & Porfiri, M. Mathematical modeling of zebrafish social behavior in response to acute caffeine administration. *Front. Appl. Math. Stat.* **7**, 751351. 10.3389/fams.2021.751351 (2021). 35493317 10.3389/fams.2021.751351 PMC9053518

[28] Syme, J., Kiszka, J. J. & Parra, G. J. Behavioural variation facilitates coexistence and explains the functions of mixed-species groups of sympatric delphinids. *Anim. Behav.* **210**, 395–408 (2024).

[29] McInnes, L., Healy, J. & Melville, J. U. M. A. P. Uniform Manifold Approximation and Projection for dimension reduction. arXiv:1802.03426v3 (2020).

[30] Bolker, B. M. et al. Generalized linear mixed models: a practical guide for ecology and evolution. *Trends Ecol. Evol.* **24**, 127–135 (2009). 19185386 10.1016/j.tree.2008.10.008

[31] Griffiths, S. W. & Magurran, A. E. Familiarity in schooling fish: how long does it take to acquire? *Anim. Behav.* **53**, 945–949 (1997).

[32] Masuda, M. et al. Identification of olfactory alarm substances in zebrafish. *Curr. Biol.* **34**, 1377–1389e7 (2024). 38423017 10.1016/j.cub.2024.02.003

[33] Akinrinade, I. et al. Evolutionarily conserved role of Oxytocin in social fear contagion in zebrafish. *Science* **379**, 1232–1237 (2023). 36952426 10.1126/science.abq5158

[34] Trompf, L. & Brown, C. Personality affects learning and trade-offs between private and social information in guppies, *Poecilia reticulata*. *Anim. Behav.* **88**, 99–106 (2013).

[35] Jolles, J., Briggs, H., Araya, Y. & Boogert, N. Personality, plasticity and predictability in sticklebacks: bold fish are less plastic and more predictable than shy fish. *Anim. Behav.* **154**, 193–202 (2019).

